# Inducing death in tumor cells: roles of the inhibitor of apoptosis proteins

**DOI:** 10.12688/f1000research.10625.1

**Published:** 2017-04-27

**Authors:** Darren Finlay, Peter Teriete, Mitchell Vamos, Nicholas D. P. Cosford, Kristiina Vuori

**Affiliations:** 1 NCI-Designated Cancer Center, Sanford Burnham Prebys Medical Discovery Institute, 10901 North Torrey Pines Road, La Jolla, CA, 92037, USA

**Keywords:** apoptosis, tumours, cancer, IAPs, inhibitor of apoptosis proteins

## Abstract

The heterogeneous group of diseases collectively termed cancer results not just from aberrant cellular proliferation but also from a lack of accompanying homeostatic cell death. Indeed, cancer cells regularly acquire resistance to programmed cell death, or apoptosis, which not only supports cancer progression but also leads to resistance to therapeutic agents. Thus, various approaches have been undertaken in order to induce apoptosis in tumor cells for therapeutic purposes. Here, we will focus our discussion on agents that directly affect the apoptotic machinery itself rather than on drugs that induce apoptosis in tumor cells indirectly, such as by DNA damage or kinase dependency inhibition. As the roles of the Bcl-2 family have been extensively studied and reviewed recently, we will focus in this review specifically on the inhibitor of apoptosis protein (IAP) family. IAPs are a disparate group of proteins that all contain a baculovirus IAP repeat domain, which is important for the inhibition of apoptosis in some, but not all, family members. We describe each of the family members with respect to their structural and functional similarities and differences and their respective roles in cancer. Finally, we also review the current state of IAPs as targets for anti-cancer therapeutics and discuss the current clinical state of IAP antagonists.

## Introduction to inhibitor of apoptosis family of proteins

**Figure 1.**
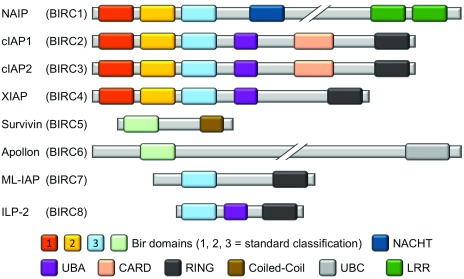
Domain structures of all known members of the human inhibitor of apoptosis protein (IAP) family, with a focus on the different baculovirus IAP repeat (BIR) domains. The representation of the homology between the different BIR domains of the IAP family reflects the accepted designation of BIR1, BIR2, and BIR3. The BIR domains of Survivin (BIRC5) and Apollon (BIRC6) can be aligned with either BIR1 or BIR2, depending on the specific alignment criteria, but owing to their uniqueness they are colored and labeled accordingly. CARD, caspase recruitment domain; cIAP1, cellular inhibitor of apoptosis protein 1; cIAP2, cellular inhibitor of apoptosis protein 2; ILP-2, inhibitor of apoptosis protein-like protein 2; LRR, leucine-rich repeat; ML-IAP, melanoma inhibitor of apoptosis protein; NAIP, neuronal apoptosis inhibitory protein; RING, really interesting new gene; UBA, ubiquitin-associated; UBC, ubiquitin-conjugating.

**Figure 2.**
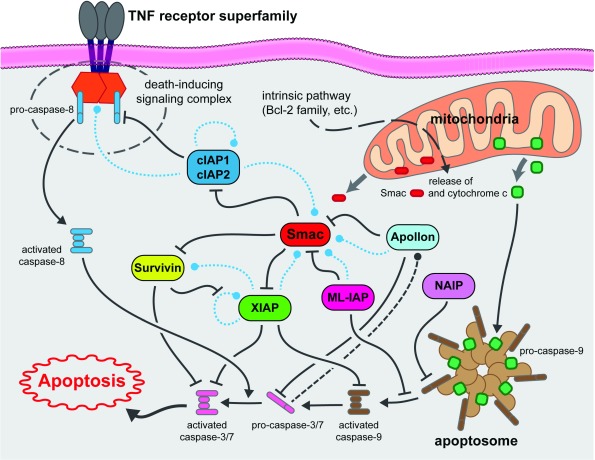
Schematic of pertinent inhibitor of apoptosis signaling pathways relevant to tumor cell survival and apoptosis. Dashed lines indicate potential degradative events (blue = ubiquitin-mediated, black = caspase-mediated). cIAP1, cellular inhibitor of apoptosis protein 1; cIAP2, cellular inhibitor of apoptosis protein 2; ML-IAP, melanoma inhibitor of apoptosis protein; NAIP, neuronal apoptosis inhibitory protein; TNF, tumor necrosis factor.

The inhibitor of apoptosis protein (IAP) family is a functionally and structurally related group of proteins that serve as endogenous inhibitors of programmed cell death, or apoptosis. In addition, some family members are regulators of another form of programmed cell death termed necroptosis ^1, 2^. Whilst some of the IAPs have also been shown to be involved in immune regulation ^3, 4^, chromosome segregation, and cytokinesis ^5– 7^, this review will focus on their roles in explicitly regulating apoptosis. Although the various IAPs have somewhat differing functions, they are linked by one unique domain: membership of the IAP family is ascribed if the gene/protein in question possesses a baculovirus IAP repeat (BIR) domain. Indeed, as the name suggests, BIR domains were first identified in a baculoviral protein capable of inhibiting cell death in virally infected cells ^8– 10^. BIR domains are zinc finger domains and invariantly contain three cysteines and one histidine, which co-ordinate the zinc ion ^10^, and these domains are involved in various protein-protein interactions (PPIs). IAPs were subsequently identified and characterized by various techniques in yeast, worms, insects, and mammalian cells ^5, 11– 15^. The first human IAP revealed was neuronal apoptosis inhibitory protein (NAIP or BIRC1), which was serendipitously discovered in a search for genes involved in the autosomal recessive condition spinal muscular atrophy (SMA) ^16^. The next human IAPs to be characterized were the cellular IAPs 1 and 2: cIAP1 (or BIRC2) and cIAP2 (or BIRC3). These proteins were discovered to have a role in tumor necrosis factor receptor (TNFR) signaling through association with the adaptor proteins TRAF1 and TRAF2 ^17– 20^. The fact that several proteins shared the common BIR domain led to the identification of more family members via traditional homology-matching database searches (reviewed in 21). Notably, many of these proteins were further shown to be involved in the regulation of apoptosis ^15, 22– 27^. Rounding out the group of eight human BIR-containing proteins are XIAP (BIRC4), Survivin (BIRC5), Apollon (BIRC6), Melanoma IAP (ML-IAP or BIRC7), and IAP-like protein 2 (ILP-2 or BIRC8) ^15, 22, 25, 28– 37^. A schematic of the general IAP family structure is shown in Figure 1. Figure 2 shows the intracellular signaling interplay of IAPs with respect to cell survival and apoptosis.

Survivin and ML-IAP are small proteins with only one BIR domain, yet their functions are enigmatic, having been ascribed to various processes, including apoptosis inhibition. As such, their roles, especially with respect to cancer, will be discussed in greater detail later in this review. ML-IAP additionally possesses an E3 ubiquitin ligase domain named RING (really interesting new gene), a domain also present in other IAPs and believed to be important in many signaling events ^38– 41^. ILP-2, likewise with only one BIR domain, also contains a RING domain and a ubiquitin-associated (UBA) domain. Whilst ILP-2 shows high homology to XIAP, it is a product of a separate gene and its expression in healthy tissues appears to be restricted to the testes ^36^. Overexpression studies have shown that ILP-2 has no effect on extrinsic death receptor-induced apoptosis but that it can inhibit intrinsic (also known as mitochondrial) apoptosis through a potential interaction with caspase-9, an apical protease involved in mitochondrial apoptosis ^36^. However, others have shown that its BIR domain is unstable and, as such, it is only a weak binder of caspase-9, at least in the absence of other cellular factors ^42^.

Apollon is an extremely large protein (approximately 528 kDa) containing only one BIR domain that is thought to be membrane-associated as well as a C-terminal ubiquitin-conjugating (UBC) domain ^43^. It has been shown to attenuate apoptosis ^44– 46^ and to directly engage and interfere with both the second mitochondria-derived activator of caspases (Smac, discussed in greater detail below) and caspase-9 ^43, 47^. Others have confirmed that Apollon is involved in caspase-9-mediated apoptosis but that it can also regulate p53 and is essential in murine embryo development ^48^.

The remaining four IAPs each possess three BIR domains in tandem and are the most studied members of the IAP family. Whilst XIAP and cIAP1 and 2 each contain a UBA and a RING domain, NAIP differs in that it has neither of these features but instead contains a “NACHT” domain and a C-terminal leucine-rich repeat (LRR). The NACHT domain is so named because of its presence in NAIP, C2TA, HET-E, and TEP1, and it is predicted to be a nucleoside-triphosphatase (NTPase) domain ^49^. Whilst its original discovery as the causative gene in SMA proved erroneous, NAIP has been shown to attenuate apoptosis in multiple models ^15, 50, 51^. The main role of NAIP, however, appears to be in the regulation of innate immunity. Thus, NAIP, which is also part of the NOD-like receptor (NLR) family, is important for NLRC4 inflammasome activation in response to certain bacterial ligands ^52– 54^.

cIAP1 and 2, whilst showing similar architecture to XIAP, also possess a caspase recruitment domain (CARD). Somewhat confusingly, however, the CARD of the cIAPs does not bind to caspases, but it appears to function in an auto-inhibitory manner to block the cIAP RING domain’s E3 ubiquitin ligase activity ^55^. The cIAPs are structurally very similar to each other with only a short linker sequence difference, are functionally redundant ^56, 57^, and are believed to have resulted from a recent evolutionary gene duplication. As with XIAP, they contain three BIR domains, and BIR1 is essential for binding to the TNFR adapter protein TRAF2 ^58, 59^. The third BIR domain (BIR3) in these three proteins, as well as the homologous BIR domain in ML-IAP, all potently bind to Smac, a negative regulator that will be discussed in much greater detail in subsequent sections of this review.

The cIAPs have been highly characterized in signaling events associated with a subset of TNFR superfamily members, called the death receptors (DRs), and it appears that their E3 ubiquitin ligase activity is especially pertinent in this regulation. DRs are categorized on the basis of the presence of a so-called death domain (DD). DDs are approximately 80-amino-acid alpha-helical structures that recruit adapter proteins capable of binding multiple other proteins in supramolecular complexes that regulate distinct signaling pathways based on composition (reviewed in 60– 63). cIAPs recruited to these complexes can be involved in both degradative K48- and non-degradative K63-branched ubiquitination. Indeed, cIAP1 has been shown to control the levels of cIAP2 via degradative signaling, as depletion of cIAP1 results in a “rebound” of cIAP2 levels ^64^. Similarly, levels of nuclear factor-kappa B (NF-κB)-inducing kinase (NIK or *MAP3K14*) are tightly controlled by cIAPs, and these protein levels are almost undetectable when the E3 ubiquitin ligase activity of the proteins is available ^64– 66^. Much more significant, however, are the cIAP-mediated non-degradative K63-branched ubiquitination and ensuing signaling. This ubiquitination of receptor-interacting protein 1 (RIP1) results in the formation of a signaling complex that can recruit further ubiquitin ligases and kinases that ultimately result in classical NF-κB activation ^67, 68^ (and reviewed in 69). Indeed, recruitment of RIP1 to these complexes has led to the coining of the term “RIPoptosome” to describe them ^70– 72^. When cIAPs are absent—owing to genotoxic stress or chemical depletion with Smac mimetics (see below), for example—and the relevant receptor agonist is engaged, RIP1 is not degraded but forms a death signaling RIPoptosome ^72^ with apoptosis effected via the apical caspases-8 or -10 or both. Furthermore, in the absence of these caspases (or upon their inhibition), necroptosis can occur ^1, 2^. Necroptosis has been demonstrated to be dependent on RIP1 and specifically on its kinase activity (reviewed in 73). RIP1 phosphorylation of RIP3 results in the activation of mixed lineage kinase domain-like protein (MLKL) ^74– 76^, which induces necroptotic death by rupturing of the plasma membrane ^77– 79^.

In sum, the cIAPs are integral components of multiple signaling complexes emanating from TNFR superfamily members and, as a consequence, can regulate diverse cellular responses such as cell survival, apoptosis, and necroptosis via the RIPoptosome ^80, 81^.

XIAP is by far the most studied and highly characterized member of the IAP family. It is a potent inhibitor of apoptosis as judged by multiple model systems and techniques and has been clearly demonstrated to effect such inhibition due to direct binding of caspases ^24^. BIR2 and a short linker section between BIR1 and BIR2 are essential for binding and sequestration of the effector caspases -3 and -7 ^24, 82– 84^, whilst BIR3 is crucial for binding to the apical caspase-9 ^85– 87^. As with the cIAPs, the BIR3 of XIAP also binds Smac, and this interaction results in caspase de-repression ^85, 88– 90^. Thus, XIAP BIR3 binding of Smac has been shown to result in the release of active caspases from the XIAP protein complex and thus BIR3-Smac interaction is permissive for apoptosis induction ^88, 91^. As such, Smac is not a direct activator of caspases, despite its name, but rather an “inhibitor of the inhibitor”. Smac effects this displacement of factors from the BIR domains because of a four-amino-acid sequence of Ala-Val-Pro-Ile (AVPI) in Smac. Exposure of cells to this peptide motif can therefore sensitize cells to apoptotic stimuli or, in the case of cIAPs, result in their auto-degradation and subsequent switch from inhibitory to pro-apoptotic events from TNFRs. Owing to these effects, the AVPI tetrapeptide sequence has drawn much attention as a potential anti-cancer agent, and multiple Smac mimetics have been developed with a view to promoting apoptosis in tumor cells, where normal apoptotic signaling is perturbed. The current clinical progress of these agents is described in detail later.

In summary, the IAP family, whilst small in number, contains a series of diverse members with differing but somewhat overlapping biological roles. The most relevant of these roles in tumors is apoptosis inhibition, and the mechanisms governing how each member is involved are somewhat unique. The next section will discuss the roles of these proteins in cancer, and finally we will discuss the application of IAP inhibitors (Smac mimetics) as potential anti-cancer agents.

## Inhibitor of apoptosis and cancer

The evasion of apoptosis is one of the hallmarks of cancer ^92– 95^, and, as noted above, the IAP family of proteins plays an important role in attenuating programmed cell death pathways, predominantly through modulation of the caspase cascade (extensively reviewed in 27, 96– 103). Furthermore, IAPs are often upregulated in cancers ^104^ and are believed to underlie the resistance of many tumors to chemotherapeutics ^105, 106^. Ablation or antagonism of IAPs is therefore an attractive strategy to sensitize or re-sensitize tumor cells to apoptosis induced by other agents. The roles that the eight IAPs found in humans play in cancer are discussed below.

### NAIP

NAIP (BIRC1) was first identified and named in 1995 by Roy *et al*. ^16^ as a potential modulator of the neuronal apoptotic pathway. As noted earlier, the main biological role for NAIP appears to be the regulation of innate immunity. Nevertheless, NAIP has been weakly linked to unfavorable prognosis in esophageal cancer ^107^, breast cancer ^108^, prostate cancer ^109^, and neuroblastoma ^110^. The precise role of NAIP in the dysregulation of apoptosis in cancer and its value as a potential therapeutic target need further study.

### cIAP1, cIAP2, and XIAP (BIRC2, BIRC3, and BIRC4)

As noted above, XIAP is a very potent binder and inhibitor of caspase-3. Accordingly, research by pharmaceutical companies has primarily focused on antagonizing this protein for oncology applications (reviewed in 111, 112). cIAP1 and cIAP2 have also been implicated in cancer, and their role in the modulation of the NF-κB signaling pathway has been investigated in detail ^113^. It was also found that cIAP1 can protect cancer cells from the lethal effect of TNF through synergy with the MYC oncogene, thus driving tumorigenesis ^114– 116^. As cIAPs suppress TNF-induced cell death, it is likely that increased levels of cIAPs support tumor cell survival by modulating cellular responses to TNF. cIAPs and XIAP are additionally thought to contribute to cancer cell invasion and metastasis through their ability to drive NF-κB-mediated expression of genes involved in cell motility, migration, and invasion ^117, 118^. Similarly, in lymphomas, cIAP2 is often found as a fusion protein with mucosa-associated lymphoid tissue 1 (MALT1), resulting in the activation of NF-κB signaling (reviewed in 119). IAPs have been shown to be overexpressed in many cell lines from the NCI60 panel as compared with the corresponding normal tissue ^120^. XIAP overexpression in turn has been reported in childhood acute myeloid leukemia (AML) ^121^, renal carcinoma ^122, 123^, multiple myeloma (MM) ^124^, and bladder cancers ^125^, and AML patients with low levels of expression of XIAP were shown to have a statistically significant survival advantage compared with those patients with higher levels ^120, 126^. In summary, the role and importance of these members of the IAP family of proteins in cancer have been extensively investigated and reviewed.

### Survivin

Survivin (BIRC5) was first identified by Altieri *et al*. as an anti-apoptosis gene expressed in various cancer cells ^25, 127^. Survivin is an example of one of the earliest IAP proteins strongly implicated in oncogenesis ^128^ and has been well established as a prognostic marker with a negative correlation on outcome in many cancers ^129– 133^ (reviewed in 134– 138). Consistent with this, excessive levels of Survivin inhibit both intrinsic and extrinsic pathways of apoptosis ^25, 139– 141^. Of note, however, Survivin is only a weak apoptosis inhibitor at physiological concentrations and may in fact exert anti-apoptotic activity through stabilization of XIAP ^142^. Recent studies have shown Survivin to be an important regulator of cell division, and this appears to be its main biological function ^5– 7, 143^. The role of Survivin in cancer has recently been reviewed extensively ^144– 147^. Therapeutic targeting of Survivin has been mostly confined to non-small-molecule strategies ^148, 149^ and repression of protein translation ^150– 152^ (reviewed in 153), and only recently have small-molecule inhibitors been reported ^154, 155^.

### Apollon

Apollon (BIRC6, the human homolog of murine BRUCE) was first identified in 1999 by Chen *et al*. ^32^ as a marker in brain and ovarian cancer cell lines that is linked to resistance to various anti-cancer drugs. A number of subsequent studies have concluded that elevated levels of Apollon are linked to poor prognosis in a range of cancers, such as leukemia ^156, 157^, breast ^158^, neuroblastoma ^159^, prostate ^160– 162^, lung ^163^, ovarian ^164^, colorectal ^165^, hepatocellular ^166^, and head and neck ^167^ cancers. It is largely understood that the role of Apollon as an oncogene is centered on its role in modulating Smac and caspase-9 levels, where overexpression of Apollon leads to increased silencing of apoptosis through Smac degradation as well as to attenuation of the caspase cascade by targeting caspase-9 for ubiquitination and subsequent degradation ^43, 45, 47^. Based on these predictive findings, the role of Apollon as a therapeutic target has been evaluated in a number of studies using functional genomic approaches, since no appropriate small-molecule tool has yet been developed ^158, 159, 166, 168^. Undoubtedly, the development of potent and selective small-molecule antagonists to Apollon will allow detailed elucidation of its potential as a therapeutic target in oncology.

### ML-IAP

ML-IAP (BIRC7, also known as Livin or KIAP) was first identified as a member of the IAP family because of its single BIR domain ^33, 35^. The ML-IAP BIR domain is also responsible for apoptosis inhibition, and small molecules that target this region could potentially re-sensitize cancer cells to chemotherapeutics. In particular, the RING domain of ML-IAP has been shown to function as an E3 ubiquitin ligase facilitating the ubiquitination and subsequent degradation of itself ^169, 170^ and, more importantly, of Smac ^170^, the natural caspase antagonist that modulates apoptotic signaling. Thus, inhibition of ML-IAP leads to a direct increase of Smac and a re-sensitization of cells to apoptotic stimuli. Both protein and mRNA levels of ML-IAP are low to undetectable in most adult tissues ^171^ but are highly expressed in several cancers ^33, 171– 179^, including various lung cancers, melanoma, liver cancer, glioblastoma, and oral squamous cell carcinoma. This protein is also highly expressed in renal cell carcinoma ^180, 181^, and this is why the original name of kidney IAP (KIAP) was coined. ML-IAP maps to chromosome 20q13, a region frequently implicated in the mutagenic etiology of lung cancers ^33^. ML-IAP levels have been shown to be highly relevant as a prognostic biomarker in lung ^172, 173, 182, 183^ and other ^174, 175, 177, 179, 180, 184– 187^ cancers. These studies have consistently reported that high ML-IAP expression correlates with a poor outcome but that lower levels predict a more favorable prognosis. A number of recent studies have clearly shown the considerable therapeutic potential of ML-IAP inhibition to treat cancer. A wealth of data has been presented in cellular contexts ^188– 195^ as well as in xenograft studies ^196, 197^. In particular, the mouse xenograft studies by Chen *et al.* ^196^ and the cell-line-based work by Zhuang *et al*. ^198^ showed a substantial benefit gained from *BIRC7* gene ablation in models of lung cancer. However, all of these studies inhibited ML-IAP through RNA knockdown approaches because of the unavailability of a selective and potent small-molecule antagonist. Recently, however, potent and uniquely selective ML-IAP inhibitors have been reported, which will help more comprehensive elucidation of the role of ML-IAP in cancers ^199^.

### ILP-2

ILP-2 (IAP-like protein-2 or BIRC8) was originally detected only in the testis and lymphoblastoid cells ^36^. However, some recent work has established a tenuous link to breast cancer ^200^, and it will be of interest to see whether this link gains further support to establish ILP-2 as a novel biomarker in human malignancies as well as a potential target for therapy.

## Inhibitor of apoptosis inhibitor development for cancer therapeutics

In the mid-1990s, it was shown that the BIR domains were necessary and responsible for the anti-apoptotic and caspase-suppressing activity of the IAP proteins ^10, 14, 84^. With the subsequent discovery of the endogenous IAP ligand Smac in 2000 ^88, 201^, the path toward the development of small-molecule inhibitors of the IAPs unfolded. Historically, however, the development of small-molecule inhibitors of such PPIs has been quite difficult. Most of these interactions are devoid of the classic druggable binding pockets (about 300–500 Å ^2^) with which most drug discovery scientists are familiar ^202^. Rather, these PPIs typically derive their binding energy from a large number of intermolecular interactions along a relatively flat and large (about 1,000–2,000 Å ^2^) surface.

**Figure 3.**
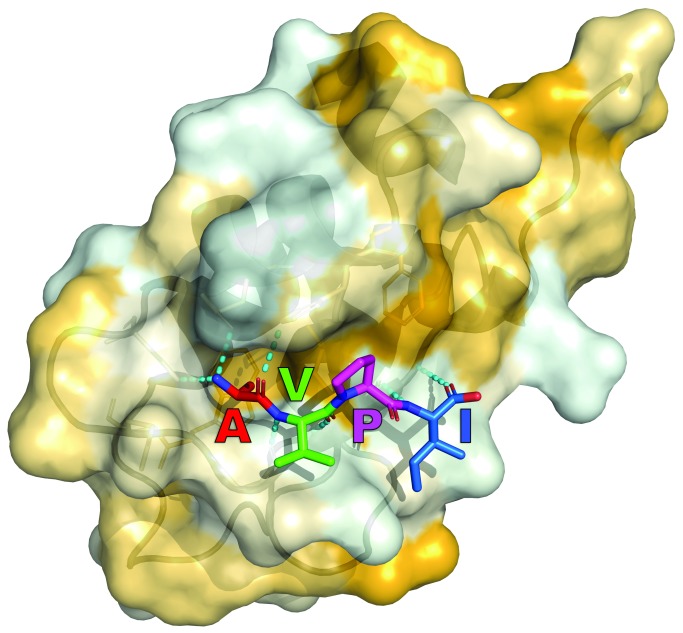
Crystal structure of Ala-Val-Pro-Ile (AVPI), a Smac core motif, bound to the BIR2 domain of XIAP (Protein Data Bank code = 4J46). Binding is strongly driven by hydrogen-bond formation (dashed cyan lines) and non-polar interactions. Hydrophobic surface properties of the BIR2 domain are shown in yellow. Note that the color scheme of the tetrapeptide sequence is maintained for the subsequent figure.

**Figure 4.**
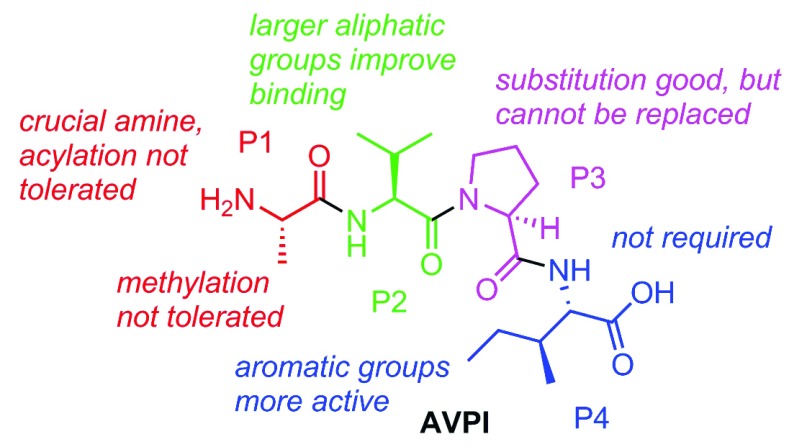
Structure-activity relationship of Smac mimetics is largely based on the original amino acid positions from the Ala-Val-Pro-Ile (AVPI) peptide.

It was a critical observation made by Xiadong Wang *et al*. regarding the loss of Smac activity upon the addition of a glutathione s-transferase (GST) fusion to its N-terminus that paved the way for the current crop of Smac mimetics ^203^. Mutation studies further confirmed the importance of the post-translationally processed and flexible N-terminus of mature Smac. Perhaps equally important was the contribution from Fesik *et al*. that year, generating the first nuclear magnetic resonance structure of truncated Smac bound to one of the IAPs, XIAP BIR3 ^89^. Specifically, four residues, AVPI, that bind to a surface groove on the IAP BIR domains proved indispensable for activity. As shown in Figure 3, there exists in the IAP BIR domains a negatively charged cleft of perfect size to accept the alanine. Furthermore, the proline of Smac allows for a crucial reverse turn feature to maintain close contacts with the binding site. These are two key elements represented in nearly all of the reported IAP inhibitors. Early on, several groups showed that synthetic oligopeptides (4–9-mers) exhibit better binding affinity than native Smac for XIAP BIR3 and are notable for their apoptosis-inducing ability ^204– 206^. These oligopeptides served an important role as a drug discovery proof-of-concept: that mimicking a small portion of Smac is a viable strategy to target the IAPs. Subsequent reports took this concept a step further and focused on developing more drug-like peptidomimetics of the N-terminal AVPI tetrapeptide binding motif to disrupt the IAP-caspase PPI, and thus far this has proven to be the most popular and successful tactic. The first true medicinal chemistry work reported by Fesik *et al*. in 2004 ^207^ laid the groundwork for the advances that would follow in subsequent years, and, also in 2004, seminal work from Wang and Harran showed that a small-molecule Smac mimetic could potentiate TNF-induced and TNF-related apoptosis-inducing ligand (TRAIL)-induced apoptosis ^208^. A summary of the collective structure-activity-relationship (SAR) conclusions from Smac mimetic medicinal chemistry work is shown in Figure 4.

**Figure 5.**
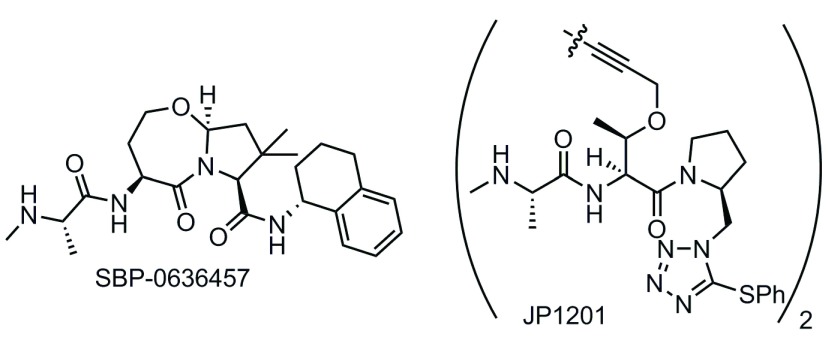
Structures of the Smac mimetic SBP-0636457 being developed by Sanford Burnham Prebys Medical Discovery Institute and the bivalent agent JP1201 from Joyant Pharmaceuticals.

A number of research groups from both academia and industry have initiated programs in the space since these early discoveries, focusing on Smac mimetics ^66, 199, 209– 256^ (also reviewed in 257, 258). Some of these compounds remain in pre-clinical testing, whereas others have entered but are no longer active in clinical trials. Our laboratories are currently testing a series of Smac mimetics developed by us at Sanford Burnham Prebys Medical Discovery Institute. A representative compound with encouraging pre-clinical data in several cancer cell lines is shown in Figure 5 ^199^. SBI-0636457 has demonstrated potent cell-killing effects in several subtypes of breast, ovarian, and prostate cancer cell lines but only when the DR ligand TRAIL or another such apoptosis inducer is co-administered. Furthermore, SBI-0636457 administered as a single agent exhibited no toxicity to normal human fibroblasts.

Bivalent Smac mimetics take advantage of the homodimeric nature of native Smac and are able to bind both the BIR2 and the BIR3 domains. The consequence of this improved binding mode is poorer drug-like properties, as the Smac mimetics must adopt a larger molecular size in order to access both binding sites. Impressive binding data (K _d_ = 300 pM for the BIR2–BIR3 construct) were observed for the first reported bivalent IAP inhibitor (JP1201) from the Wang and Harran labs ( Figure 5) ^208^.

## Current clinical status of inhibitor of apoptosis inhibitors in oncology

**Figure 6.**
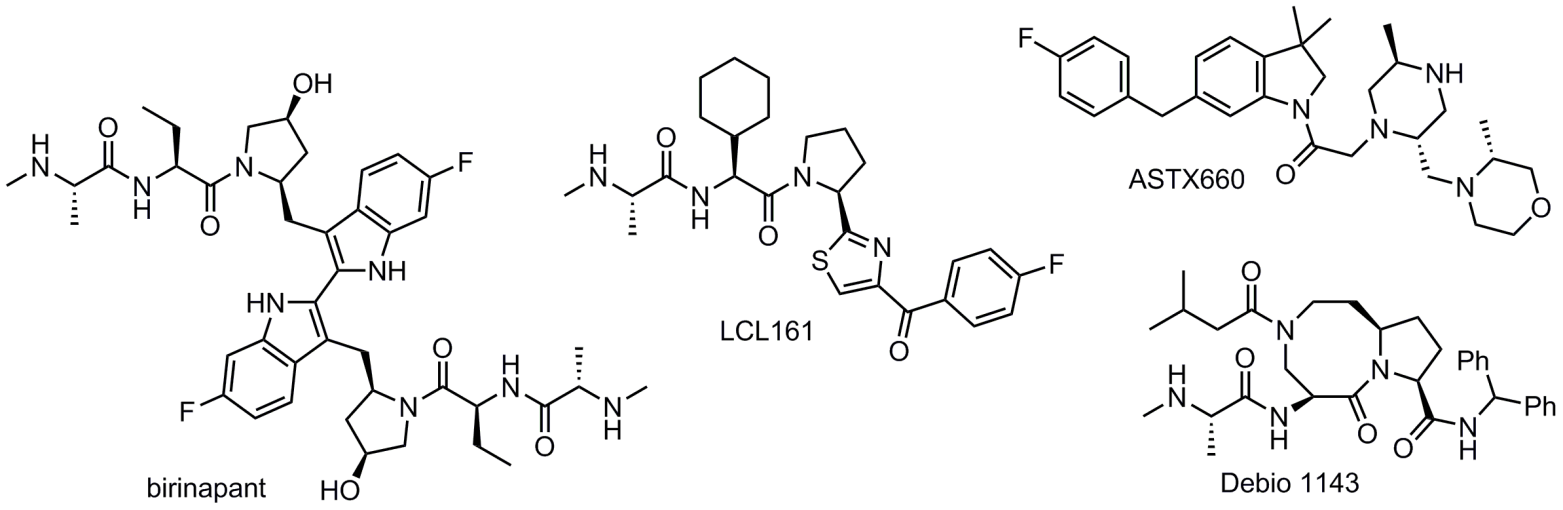
Chemical structures of inhibitor of apoptosis inhibitor compounds in active clinical trials.

In the US, several monovalent Smac mimetic compounds and one bivalent compound have entered the clinic and are still active in clinical trials ( Figure 6). All of the compounds for which clinical data have been reported so far demonstrated generally favorable safety profiles in phase I, and amylase/lipase elevation, alanine and aspartate transaminase (ALT and AST) elevation, cytokine release syndrome (CRS), and Bell’s palsy were the dose-limiting serious adverse events ^112^. Of note, however, the Bell’s palsy toxicity has been observed only with bivalent and not with monovalent Smac mimetics. It has been suggested that CRS may result from the Smac mimetic-induced degradation of cIAP1 and the consequent activation of the NF-κB pathway and an autocrine/paracrine TNF signaling loop. Other possibilities exist, however, as work from Silke and Vaux suggests that triple knockdown of cIAP1, cIAP2, and XIAP results in a hyperactive inflammatory state through still-undefined mechanisms (reviewed in 259). While TNF release potentially enables the efficacy of Smac mimetics as single agents in cancer therapy, the possibility of inducing a “cytokine storm” may render this approach less desirable compared with a combination approach (TNFR agonists + Smac mimetics), especially for indications outside of cancer ^260, 261^. Indeed, Smac mimetics have demonstrated synergy with other modes of treatment, including cytotoxic agents (that is, carboplatin ^262^ and paclitaxel ^263^), radiation therapy ^264^, and cell DR ligands (TRAIL analogues) ^265^. These synergies are well defined in pre-clinical models, but, so far, they have been less successful in clinical settings (see below). In general, any treatment that stresses the cells, such as standard chemotherapy or radiation therapy, and induces either intrinsic or extrinsic apoptosis via upstream activation could be combined with the caspase-liberating effect of IAP inhibitors to kill cancer cells. Although a number of Smac mimetics have already entered clinical trials, we shall focus our discussion here on those for which trials are currently active ( Table 1).

**Table 1.** Ongoing clinical trials with inhibitor of apoptosis inhibitors.

NCT number	Phase	Title	Interventions	Conditions	Sponsor
02587962	I/II	Dose escalation study of birinapant and pembrolizumab in solid tumors	Birinapant, pembrolizumab	Solid tumors	TetraLogic (Medivir)
02756130	II	Proof-of-concept study of birinapant in combination with platinum-based chemotherapy in subjects with high-grade serous carcinomas	Birinapant, paclitaxel, carboplatin	Advanced newly diagnosed or recurrent high-grade serous carcinomas	TetraLogic (Medivir)
01486784	I/II	A phase I-II open-label non-randomized study using TL32711 for patients with acute myelogenous leukemia, myelodysplastic syndrome, and acute lymphoblastic leukemia	Birinapant	Acute myelogenous leukemia	Tetralogic (Medivir)
01934634	I	Phase I trial of LCL161 and gemcitabine plus nab-paclitaxel in metastatic pancreatic cancer	LCL161, gemcitabine, nab-paclitaxel	Metastatic pancreatic cancer	Novartis
01955434	II	SMAC mimetic LCL161 alone or with cyclophosphamide in treating relapsed or refractory multiple myeloma	LCL161, cyclophosphamide	Recurrent and refractory plasma cell myeloma	Mayo Clinic
02649673	I/II	LCL161 plus topotecan for patients with relapsed/ refractory small cell lung cancer and select gynecologic malignancies	LCL161, topotecan	Small cell lung cancer, ovarian cancer	Novartis
02098161	II	Phase II LCL-161 in patients with primary myelofibrosis, post-polycythemia vera myelofibrosis, or post-essential thrombocytosis myelofibrosis	LCL-161	Leukemia	Novartis
02890069	I	A study of PDR001 in combination with LCL161, everolimus, or panobinostat	LCL161, PDR001, everolimus, panobinostat	Colorectal cancer, non-small cell lung carcinoma, triple- negative breast cancer	Novartis
02022098	I/II	Debio 1143-201 dose-finding and efficacy phase I/II trial	Debio 1143, cisplatin, radiotherapy	Squamous cell carcinoma of the head and neck	Debiopharm
02503423	I/II	Phase I-II study of ASTX660 in subjects with advanced solid tumors and lymphomas	ASTX660	Solid tumors, lymphoma	Astex

### Birinapant

Birinapant is a bivalent Smac mimetic developed by Tetralogic Pharmaceuticals and currently owned by Medivir. Owing to the size of this molecule, only administration by intravenous line has been reported for birinapant, both as a single agent and in combination with several chemotherapeutics (azacitidine, gemcitabine, irinotecan, and conatumumab) ^266^. The data released so far have been lackluster, and poor efficacy has been demonstrated in the completed studies. In NCT01681368, no complete nor partial response was observed in solid tumors of 11 patients, and accrual was terminated for lack of detected clinical benefit ^267^. Good, though muted, news came in the phase I/II trial NCT01188499. In patients with metastatic colorectal cancer who previously failed irinotecan treatment, the combination of irinotecan with birinapant resulted in disease stabilization in 62% of patients, higher than the 41% rate shown by the recently approved kinase inhibitor regorafenib ^268^. The other silver lining for these results is that the response rate as measured by tumor regression was higher, albeit small, at 8% compared with regorafenib at 1% ^269^ and that the enrolled patients had previously failed all available treatments. When comparing these two sets of trial data, one must bear in mind that the regorafenib data come from an earlier and much larger phase III trial. The failure of birinapant versus placebo in a study (NCT02147873) investigating its capacity to treat myelodysplastic syndrome resulted in the folding of Tetralogic and transfer of assets to Medivir, where trials are ongoing ^270^. Given the previous failures, it will be critical to see favorable clinical outcomes for the conatumumab (DR5 agonist) combination therapy trial (NCT01940172) as well as the trial with pembrolizumab (PD-1 inhibitor) as co-treatment (NCT02587962). CRS has so far proven to be a relatively minor adverse event in patients who received birinapant, and only 10% of patients reported low-grade symptoms ^266^.

### LCL161

LCL161 is a monovalent IAP inhibitor developed by Novartis that is currently in active clinical trials and has also shown generally good safety up to a 1,800 mg dose; CRS was the major adverse event ^271^. Despite the encouraging safety profile, early results in a phase I trial (NCT01098838) indicate no objective response from LCL161 single-agent treatment in patients with solid tumors, and the best response was stable disease observed in 19% of patients. These early trial data indicate that the use of Smac mimetics as a monotherapy may be limited by the amount of CRS elicited by the drug or, more broadly, the class of drugs ^272^. Results from the phase II study (NCT01617668) testing LCL161 in combination with paclitaxel in triple-negative breast cancer indicate that this approach may circumvent the CRS issues, as it was a serious adverse event for only 0.94% of patients ^273^. It must be noted that it is not clear why the LCL161 plus paclitaxel treatment did not elicit the same CRS response as observed in the phase I study of LCL161 single-agent treatment at the same 1,800 mg dose. The study also revealed a 38% pathological complete response rate versus 17% for paclitaxel alone in a select patient population (for details of the study design, see 274). Interestingly, the increased pathological response rate is observed in a subgroup of patients who showed an elevated TNF-alpha/RIP1 gene signature prior to treatment. These data are encouraging and also help to further the idea of the need for a combination therapy with Smac mimetics. More recently, LCL161 has been tested in combination with cyclophosphamide in MM (NCT01955434). The combination of cyclophosphamide with LCL161 resulted in progression-free survival of 10 months in patients with relapsed/refractory MM ^275^. LCL161 was also shown to be effective in a transgenic mouse myeloma model, appearing to act via an immunological mechanism ^275^. Chesi *et al.* ^275^ demonstrated that the antagonism of IAPs by LCL161 does not result in direct killing of tumor cells, but rather it induces a tumor-cell autonomous type 1 interferon response. This results in a strong inflammatory response that ultimately leads to phagocytosis of the cancer cells. Intriguingly, the authors further show that LCL161 combination with PD-1 blockade was curative of all mice that completed 2 weeks of treatment ^275^. Expanding on these findings is recent evidence that immune checkpoint blockade combined with Smac mimetics is efficacious in pre-clinical models of glioblastoma ^276^. As such, the immune regulatory roles of IAPs may also be of much therapeutic relevance.

### Debio 1143

Debio 1143 is another monovalent Smac mimetic in ongoing clinical trials for a number of different malignancies. It was developed in its early stages by the Wang group at the University of Michigan and later at Ascenta Therapeutics, ultimately being licensed to Debiopharm. Phase I safety studies were in line with the previously reported Smac mimetics reported above, when tested as a monotherapy: generally mild adverse events with a highest tested dose of 900 mg ^277^. On-target pharmacodynamic modulation was achieved at doses above 80 mg, as measured by cIAP1 degradation. Preliminary efficacy data from the trial indicated that 20% of patients exhibited stable disease as the best response. With the encouraging safety data, a phase I/II trial (NCT02022098) with cisplatin and radiotherapy as co-treatment was undertaken for squamous cell carcinoma of the head and neck, and the expected completion date is 2019.

### ASTX660

UK-based Astex Pharmaceuticals recently initiated their own phase I/II trial (NCT02503423) for the small-molecule ASTX660 for solid tumors and lymphomas. Envisioning ASTX660 as part of a two-pronged cell death approach, Astex screened a number of breast, colorectal, ovarian, leukemia, and melanoma cell lines for their response to monotherapy versus co-treatment with TNF-alpha ^278^. It will be interesting to see how well the pre-clinical data correlate with the clinical data expected in 2018.

While the ability of the reported Smac mimetics to induce cancer cell death in pre-clinical models was exciting and held much promise, so far the first-in-human studies have presented lackluster results. Several compounds that were able to induce cancer cell death and thus partial or complete remission in tissue culture and animal studies have not had similar success in trials as a monotherapy. However, given the promising clinical pharmacodynamics and safety data, further research and development efforts are certainly warranted.

## Conclusions and future work

As detailed above, the IAPs are at the nexus of cancer cell survival and, conversely, apoptosis. As such, the inhibition of pertinent family members would be expected to afford a valuable therapeutic intervention strategy for cancers, as these diseases are largely conditions of increased proliferation and impaired apoptosis. As often occurs, however, the reality has proven vastly more complicated than first envisioned. As detailed above, although Smac mimetics are safe and well tolerated, they have shown little single-agent activity in clinical trials. Intuitive, yet not extensively pre-clinically verified, combinations of IAP antagonists such as Smac mimetics with standard-of-care chemotherapeutics have likewise proven unfruitful to most degrees, although there have been some responses, as described above. Perhaps most encouraging have been pre-clinical studies showing that IAP antagonists are potent sensitizers to certain TNFR family agonists ^64, 199, 208, 279– 283^. Additionally, it has been shown that this can be effected not only by the natural ligands themselves but also by agonistic antibodies to TRAIL receptors developed by several pharmaceutical companies ^284– 288^. Targeting TRAIL receptors with simultaneous IAP inhibition not only is toxic to cancer cells but also leaves non-transformed cells untouched, a “holy grail” of anti-cancer therapy. Expanding on these observations are studies by Beug *et al*., who show that concomitant induction of an immune response when IAPs are inhibited can produce a profound tumor regression in animal models ^289^. Indeed, the use of Smac mimetics and attenuated oncolytic viruses as an anti-cancer strategy has shown promising results in some models ^290^. As such, the notion of targeted activation of certain TNFRs in combination with IAP inhibition is a potential potent intervention point in many cancers. Already, clinical trials of just such a combination are underway (discussed above), and the results should further assist in our progress toward more targeted therapies using these phenomena.

In sum, whilst the clinical application of IAP antagonists has to date not produced the panacea desired, the ongoing development of next-generation agents and pertinent combinations bodes well for the future. “Inhibiting the inhibitors” ^291^ may soon be a viable anti-cancer strategy.
